# CircC16orf62 promotes hepatocellular carcinoma progression through the miR-138-5p/PTK2/AKT axis

**DOI:** 10.1038/s41419-021-03866-7

**Published:** 2021-06-09

**Authors:** Shuai Zhang, Yuan Lu, Hong-Yu Jiang, Zhi-Mei Cheng, Zi-Jing Wei, Yun-Hao Wei, Ting Liu, Bai-Juan Xia, Xu-Ya Zhao, Yu Huang, Xun Zou, Rong Liu, Shi Zhou

**Affiliations:** 1 Department of Interventional Radiology, The Affiliated Cancer Hospital of Guizhou Medical University, Guiyang, 550000 P.R. China; 2 Department of Interventional Radiology, the Affiliated Hospital of Guizhou Medical University, Guiyang, 550004 P.R. China; 3 Department of Cancer Research Laboratory, The Affiliated Cancer Hospital of Guizhou Medical University, Guiyang, 550000 P.R. China; 4 Guizhou Provincial Key Laboratory of Pharmaceutics, Guizhou Medical University, Guiyang, 550004 P.R. China; 5 School of Basic Medical Sciences, Guizhou Medical University, Guiyang, 550004 P.R. China; 6 Department of Radiology, the Affiliated Hospital of Guizhou Medical University, Guiyang, 550004 P.R. China; 7 Department of Interventional Radiology, First Affiliated Hospital of Guizhou University of Traditional Chinese Medicine, Guiyang, 550000 P.R. China

**Keywords:** Cancer, Cell growth

## Abstract

Circular RNA (circRNAs) functions vital in the pathogenesis and progression of hepatocellular carcinoma (HCC). However, the expressions and functions of certain circRNAs on metastasis and proliferation of that cancer is still unclear. Bioinformation analysis and qRT-PCR indicated that CircC16orf62 was prominent upregulated in HCC of which the expression level was positively associated to cancer’s malignant progression. Gain or loss-of-function studies indicated that the reduction of CircC16orf62 expression promotes the proliferation, invasion, and glycolysis of HCC in vitro and in vivo. The bioinformatic analysis found that miR-138-5p and PTK2 were the downstream target of CircC16or62. Then, the FISH(Fluorescence immunoin situ hybridization) and cell nucleoplasmic separation determined that CircC16orf62 located in the cell cytoplasm. Plasmid vectors or siRNAs were used to change the expression of CircC16orf62, miR-138-5p, and PTK2 in PC cell lines. CircC16orf62 functioned as a molecular sponge for miR-138-5p, and a competitive endogenous RNA for PTK2, promoting AKT/mTOR pathway activation. Our observations lead us to conclude that CircC16orf62 functions as an oncogene in HCC progression, behaving as a competitive endogenous RNA for miR-138-5p binding, thus activating the AKT/mTOR pathway. In conclusion, CircC16orf62 is an oncogene through the miR-138-5p/PTK2/Akt axis in HCC cells, indicating CircC16orf62 can be a therapeutic target with potentiality for liver cancer and a predictive marker for people with HCC.

## Introduction

Ninety percent of patients with primary liver carcinomas suffer hepatocellular carcinoma (HCC)^1^ which possesses high morbidity and mortality rates, causing malignancy. Although vital achievements are made in therapies for HCC, the 5-year livability stays low. What needs to mention is that, the molecular pathogenesis and therapeutic targets of the cancer is still unclear. So it is obvious that we need to understand its pathogenic process and regulatory mechanisms. RNAs regulate cell activities including circular RNAs, microRNAs, and other noncoding RNAs^2^.In the 1970s, circular RNA was first discovered and confirmed in a virus^3^. For the limits of detection, we viewed circRNA as a situation of incorrect splicing while in exon transcription^4^. So the existence had not got enough attention^5^. Because of its special molecular biological structure, circular RNA has been ignored by biologists, although it may play an extremely critical role^6^. Expression of CircRNAs can be found widely in human cells with various expression levels in different tissues^7^. Researches suggest that malignancy is regulated by circRNAs, such as the proliferation of cancer cells and etc^8^. Those researches also investigated the function of circRNAs in cancers^9^. For instance, the proliferation and migration of cervical cancer cells is regulated by circCLK3 which modulates the FoxM1 gene expression by sponging miR-320a^10^. The proliferation of gastric cancer and esophageal carcinoma cells were promoted by the CircAmotl1 via the host gene Amotl1 which can be considered as an oncogenic factor^11^. CircRNAs sponge the target miRNAs like competing endogenous RNAs (ceRNAs). Recently, it is been reported that the proliferation and invasion of esophageal squamous cell carcinoma can be improved by FUT8 which helps sponge miR-570-3p to promote Krupple-like Factor 10^12^. The circRNA AKT1 can sponge miR-942-5p to affect the AKT1 expression, by enhancing the cervical cancer progression^13^. Also, the RNA type we study here interacts with transcription factors and proteins to build and regulate circRNPs. Circular RNA circCUL3 was revealed that combined with STAT3 to accelerate the Warburg effect progression of gastric cancer through regulating the HK2 expression^14^. The current study indicates that circRNAs function vital for the initiation, migration, and invasion of HCC^15,16^, but the mechanism on regulating malignancy remains unclear^17^.

Here we examined the differentially expressed circRNAs which noticeably downregulated in HCC tissues and figured out circC16orf62 over-expression significantly attenuated its malignancy. And we investigated the possible targeted miRNAs and noticed that circC16orf62 sponges oncogenic miR-138-5p like a ceRNA, enhancing oncogene PTK2 expression and attenuating the malignancy.

## Materials and methods

### HCC specimens

The experiment here got approval from the Ethics Committee of Guizhou Medical University. Eighty-eight pairs of surgical or biopsied HCC and adjacent non-tumor specimens in 2015 from 2020 were obtained and they were gained with the informed consent of patients. Diagnoses of patients are according to the practice guidelines of the American Association for the Study of Liver Diseases (AASLD). Tissue RNA was gained by RNeasy Mini Kit (Qiagen, Hilden, Germany), and measurement on RNA concentrations was by NanoDrop 2000 spectrophotometer (ThermoScientific, Waltham, MA). The contained circRNAs were enriched and digested with RNase A and reversely transcribed into cRNA by fluorescent reagents and random primers. This study was approved by the Ethics Committee of The Affiliated Cancer Hospital of Guizhou Medical University.

### Cell culture

Human HCC BEL7402, QGY7701, SMMC7721, Huh-7, Hep3B, HepG2, non-tumor human liver cells were got from American Type Culture Collection (ATCC, Manassas, VA, USA), culturing in DMEM and RPMI-1640 containing 20% fetal bovine serum (FBS),100 units/ml of penicillin, and 100 µg/ml of streptomycin at 37 °C in an incubator of 5% CO_2_.

### siRNA and plasmid transfection

Shanghai Hanheng Biotech (Shanghai, China) provided the plasmid of CircC16orf62-shRNA, miR-138-5pmimic, and the two’s negative control miRNAs. The siRNA, miRNA mimic, and miRNA inhibitor were transfected into the HCC cell used by Lipofectamine 3000 (ThermoFisher). The leti-virus plasmid was transfected into the HCC cells with polybren. And the transfection lentivirus content was according to the individual multiplicity of infection (MOI) in the presence of 5 µg/ml puromycin.

### Polymerase chain reaction

We collected different cells and RNAs extraction by Trizol reagent (Invitrogen). Superscrpt II kit (Invitrogen) reversely transcribes the RNA we got into cDNA. The evaluation on relative expression levels of target gene RNA transcripts were got by qRT-PCR of SYBR Green mix (Takara, Dalian, China).

### Western blot

Protein samples isolated from indicated pancreatic cancer cells were loaded onto SDS-PAGE and transferred to PVDF membranes. Membranes were blocked with 5% skimmed milk, incubated with primary antibodies HK2 (Anti-HK2, 1:1000, ab209847, Abcam), PKM2 (Anti-PKM2, 1:1000, ab85555, Abcam), GUT1(Anti-GUT1, 1:1000, ab115730, Abcam), PTK2 (Anti-PTK2, 1:1000, ab40794, Abcam), AKT(Anti-AKT, 1:1000, ab18785, Abcam), p-AKT(Anti-AKT1-phospho-S473, ab81283, Abcam), mTOR(1:1000, 66888-1-Ig, Proteintech), p-mTOR(Anti-mTOR-phospho S2448, ab109268, Abcam), GAPDH (1:1000, 60004-1-Ig, Proteintech), followed by reaction with HRP-conjugated secondary antibodies (1:1000, Boster). ECL Western Blotting Substrate (Beyotime) was applied for Western blot band detection. Images were visualized under Biorad Imaging Systems.

### Luciferase assay and dual-luciferase reporter system

The plasmid psiCHECK2 contains the cloned CircC16orf62 WT and its mutant sequences. HCC cells (4 × 10^4^ cells/well) were cultivated in 6-well plates overnight and transfected with psiCHECK vector, psiCHECK-CircC16orf62 WT, psiCHECK-CircC16orf62 mutant, with the plasmid for Renilla luciferase expression by lipofectamine 3000. After 24 h, the cells were lysed and luciferase activities were investigated by Dualluciferase reporter assay system (Promega, US).

### glucose consumption, lactate production, cellular ATP level

Glucose consumption and lactate production were detected with the assay kit purchased from Beyotime(Shanghai, China). The glucose and lactate levels in the medium were measured using a glucose assay kit and a lactate assay kit according to the instruction manual respectively. The level of glucose and lactate were normalized to corresponding cells number. For the cellular ATP level production was analyzed by the CellTiter-Glo 2.0 Assay kit according to the manufacturer’s instructions. The relative intracellular ATP level was normalized to the cell number of corresponding cell lysate. The Seahorse XF-96 Wave software was used to analyze the data. All values were normalized to cell numbers.

### Transwell invasion assay

Transwell invasion assay was used for determining the invasion of different groups of HCC cells (10^5^ cells/well) which were cultivated in serum-free medium in the top chamber that had been loaded with Matrigel. Seven hundred microliters of complete medium was in the bottom chambers. Twenty-four hours later, the cells on the upper surface of the top chamber were removed with a cotton swab was used for taking the cells away from the upper surface of the top chamber, then the cells on the top chamber were dyed by Harris purple crystal solution (0.2%) (Sigma) and photoimaged.

### In vivo assay

For the in vivo assay, the HCC cells of circC16orf62 knockdown, negative control were subcutaneously injected into BALB/c nude mice (4–6-weeks old, female, *n* = 5 per group). The nude mice were observed every week and euthanized in 5 weeks after injection. The tumors were dissected and weighed. all conditions and procedures for the animal experiments were approved by the Animal Care Committee of The Affiliated Cancer Hospital of Guizhou Medical University.

### Statistical analysis

All the data were analyzed and performed using GraphPad prism 8.0 (La Jolla, CA, USA) or SPSS Statistics software (Armonk, NY, USA). Two groups were estimated using a student’s *t*-test, and three groups and more than three groups were using the non-parametric test (Mann–Whitney test) or One-way ANOVA test. The overall survival rate was calculated according to the Kaplan–Meier analysis. *p ≤ 0.05, **p ≤ 0.01, and ***p ≤ 0.001 were considered statistically significant.

## Results

### CircC16orf62 is upregulated in HCC and associated with progression

**Fig. 1:**
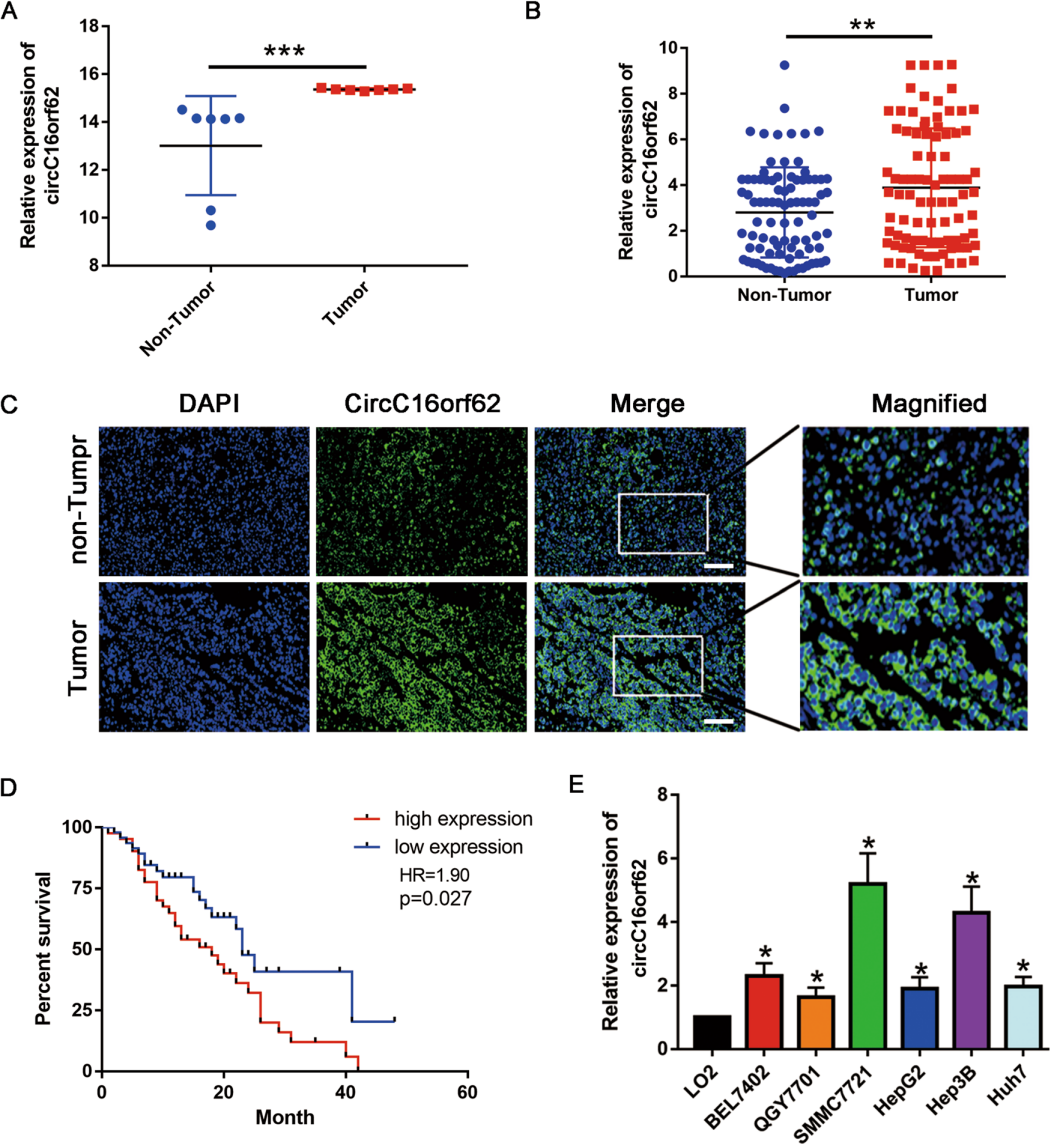
CircC16orf62 is upregulated in HCC and associated with progression. **A** Bioinformation analysis the circC16orf62(circRNA_0005699) expression in the HCC tissues and adjacent tissue based on the GEO database (GSE97332). **B** q-PCR analysis the expression level of circC16orf62 in tumor tissues and adjacent normal tissues. **C** FISH assay was used to examine the expression of circC16orf62 in the HCC tissue, the scale bar represents 50 µm. **D** Kaplan–Meier analysis showed that the expression of circC16orf62, was predictive of overall survival in HCC. **E** qRT-PCR to detect the expression of circC16orf62 in six human HCC cell lines and one human non-cancerous cell line (LO-2). Data are shown as the mean ± standard deviation of three independent experiments.*P < 0.05 was regarded as statistically significant.

We downloaded the Chip data in the GEO database for screening the differently expressed circRNAs in the HCC tissues and adjacent tissue. The results indicated that the expression of circC16orf62(circRNA_0005699) was higher in tumor tissues than in adjacent normal ones which can be seen from Fig. 1A. To solidify results, 88 pairs were studied the expression level of circC16orf62 by qRT-PCR. It is indicated that circC16orf62 was overexpressed in the HCC tumor tissue than in adjacent normal tissues (Fig. 1B). The FISH assay evaluated the expression, the result indicated that the expression of circC16orf62 was higher in the HCC tissue than in the adjacent tissue (Fig. 1C). Then, survival analysis assessed the circC16orf62 value in diagnose and we found that circC16orf62 expression was negatively associated to prognosis (Fig. 1D). The measurement was performed on circC16orf62 in the healthy liver epithelial cell line LO2 and in 7 HCC cell lines, including BEL7402, QGY7701, SMMC7721, HepG2, Hep3B, Huh7. This circRNA expressed much higher in human HCC cell lines than in normal liver epithelial (Fig. 1E). Table 1 indicates the relation between the expression and clinical parameters. No obvious distinguishment in the expression of this circRNA between the two groups when taking age, gender, cirrhosis or HBV virus infection into consideration. The expression was vitally associated to Diameter of tumor (*p* < 0.05) and lymph node metastasis (*p* < 0.05). The above suggested this circRNAs was upregulated in HCC and may related to tumorigenesis and its advance.

**Table 1:** Correlation between circC16orf62 expression and clinicopathological characteristics of HCC patients.

Clinical epidemiology and clinicopathologic feature		circC16orf62		*p* value
		High	Low expression	
All cases		56	32	
Age				
	≤50	21	17	0.183
	>50	35	15	
Gender				
	Male	31	12	0.125
	Female	25	20	
Diameter of tumor				
	≤4	25	23	0.016*
	>4	31	9	
HBV virus infection				
	Negative	24	18	0.271
	Positive	32	14	
Cirrhosis				
	Negative	16	11	0.634
	Positive	40	21	
Distant metastasis				
	Negative	45	27	0.777
	Positive	11	5	
Lymph node metastasis				
	Negative	39	29	0.033*
	Positive	17	3	
TNM stage				
	I/II	42	27	0.421
	III/IV	14	5	

*p values that are statistically significant are shown in bold.

### The characteristics of the circC16orf62

**Fig. 2:**
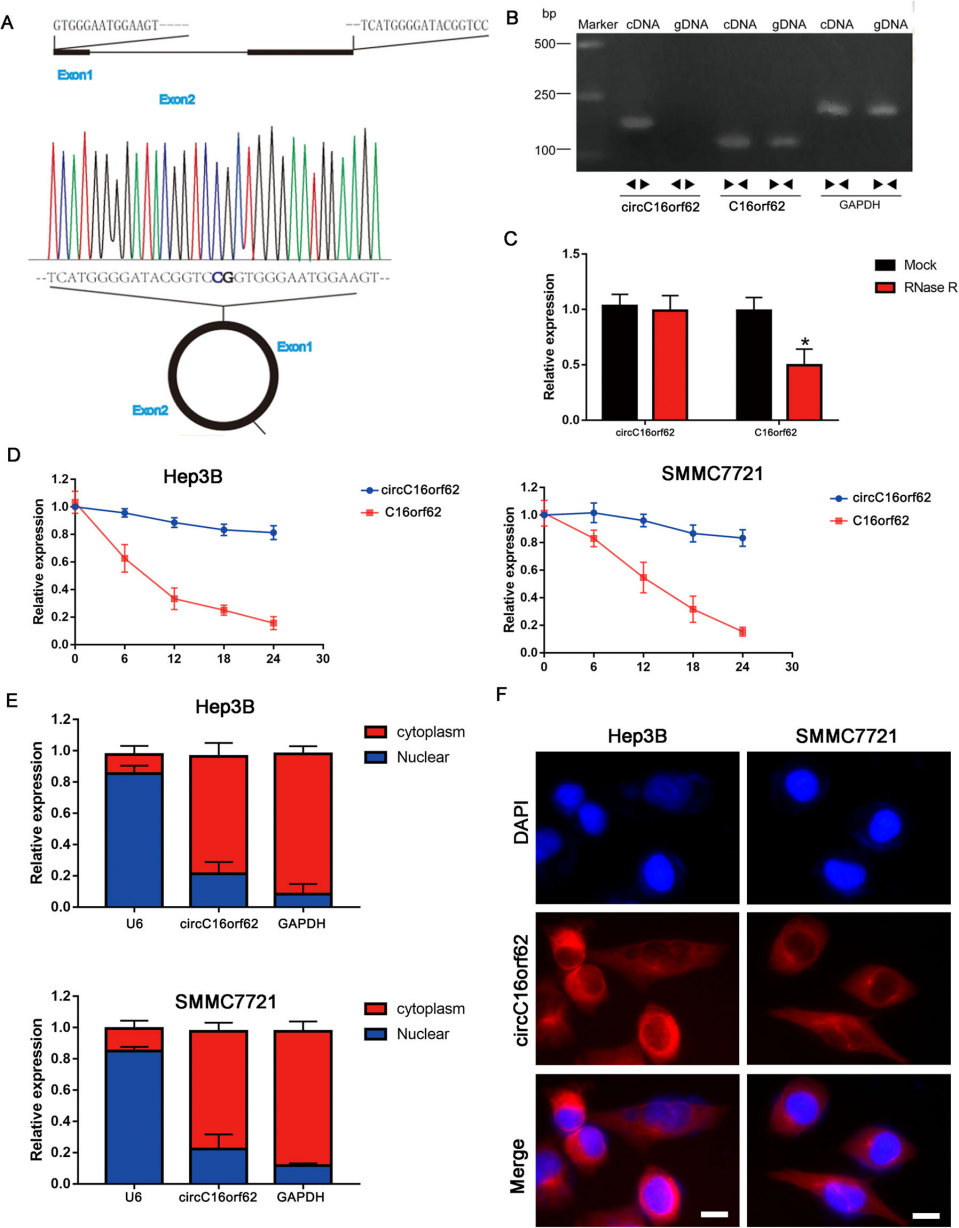
The characteristics of the circC16orf62. **A** circUBA1 originates from back-spliced exons 1 to 2 of C16orf62. The back-splice junction of circC16orf62 was identified by Sanger sequencing. **B** PCR analysis for circC16orf62 and linear C16orf62 in cDNA and gDNA. **C** The stability of circC16orf62 was evaluated by RNase R digestion assay. **D** PCR analysis for circC16orf62 and linear C16orf62 in cDNA and gDNA by RNase R digestion assay. **E** Nucleoplasmic separation analysis the location of circC16orf62. **F** FISH assays showed that circC16orf62 was distributed principally in the cytoplasm, the scale bar represents 10 µm. Data are shown as the mean ± standard deviation of three independent experiments. *P < 0.05was regarded as statistically significant.

Investigation on the characteristics of circC16orf62 need to be carried out before delving into the speciality of that in HCC. Figure 2A shows the genomic locus of circC16orf62. The spliced mature sequence length is 1198 bp. For that head-to-tail splicing is the result of reverse splicing of cDNA and genomic rearrangements, convergent primers and divergent primers were created for circC16orf62 to perform PCR using cDNA or genomic DNA from SMMC7721 cells as the template. Fig. 1C shows that circC16orf62 was amplified by the divergent primers only from cDNA but not from gDNA. Further, RNase R exonuclease and actinomycin D examine the authenticity of circC16orf62 in Hep3B and SMMC7721 cells. Figure 2C, D shows that circC16orf62 was resistant to RNase R and actinomycin D, whereas C16orf62 line RNA was significantly inhibited after RNase R and actinomycin D treatment. The cytoplasmic nuclear isolation experiment and RNA FISH assays illustrated that most of the circC16orf62 can be found in the cytoplasm (Fig. 2E, F). Data above illustrated that the circC16orf62 samples was indeed circular and mainly located in the cytoplasm.

### circC16orf62 promotes the proliferation, metastasis, and glycolysis of HCC cells

**Fig. 3:**
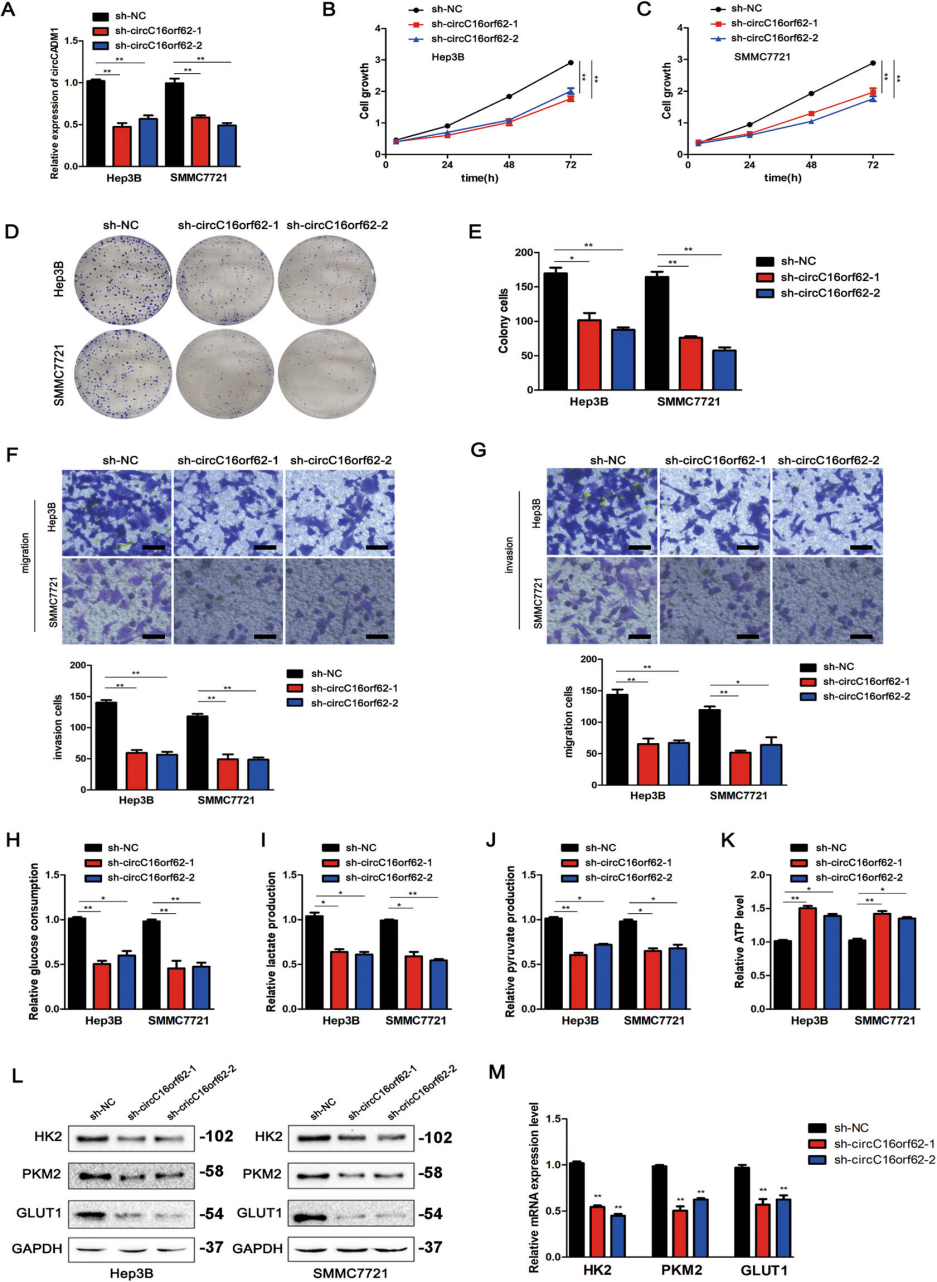
circC16orf62 promotes the proliferation, metastasis, and glycolysis of HCC cells. **A** The effects of knockdown or overexpression of circC16orf62 in Hep3B or SMMC7721 cells were measured using qRT-PCR. **B**–**E** CCK-8 and colony formation assays of PC cells with circC16orf62 knockdown. **F**, **G** Transwell assays showed that invasion and migration were significantly inhibited after circC16orf62 knockdown, the scale bar represents 50 µm. **H**–**K** Glucose consumption (**H**), lactate production (**I**), pyruvate production (**J**), ATP production (**K**) were measured in circC16orf62 knockdown HCC cells. **L**, **M** RT-qPCR and western blot analysis several metabolic enzymes (GLUT1, PKM2, HK2) expression level were in circC16orf62 knockdown HCC cells. Data are shown as the mean ± standard deviation of three independent experiments. *P < 0.05 was regarded as statistically significant.

To found the biological role of circC16orf62 in HCC cells, we constructed the circC16orf62 shRNA and transfected the plasmid into the HCC cells Hep3B and SMMC7721. qRT-PCR verifies the transfection efficiency (Fig. 3A). Then, we used CCK-8 and plate colony assays to find the proliferation ability and evaluated the invasion and migration of the HCC cells by the transwell assays. Fig. 3B–E indicated that knockdown of circC16orf62 noticeably inhibited both the proliferation, Fig. 3F, G shows the invasion and migration ability of HCC cells. Glucose consumption, lactate production, pyruvate production, and ATP level were examined in circC16orf62 downregulated and their control respectively. circC16orf62 can increase glucose consumption (Fig. 3H), lactate production (Fig. 3I), pyruvate production (Fig. 3J), and decrease ATP level (Fig. 3K). Then, the western blot and q-PCR indicated the aerobic glycolysis enzymes were downregulated in the circC16orf62 knockdown group. These results implied that circC16orf62 could promote proliferation, metastasis, and aerobic glycolysis in HCC cells.

### MiR-138-5p was the potential target of circC16orf62 in HCC cells

**Fig. 4:**
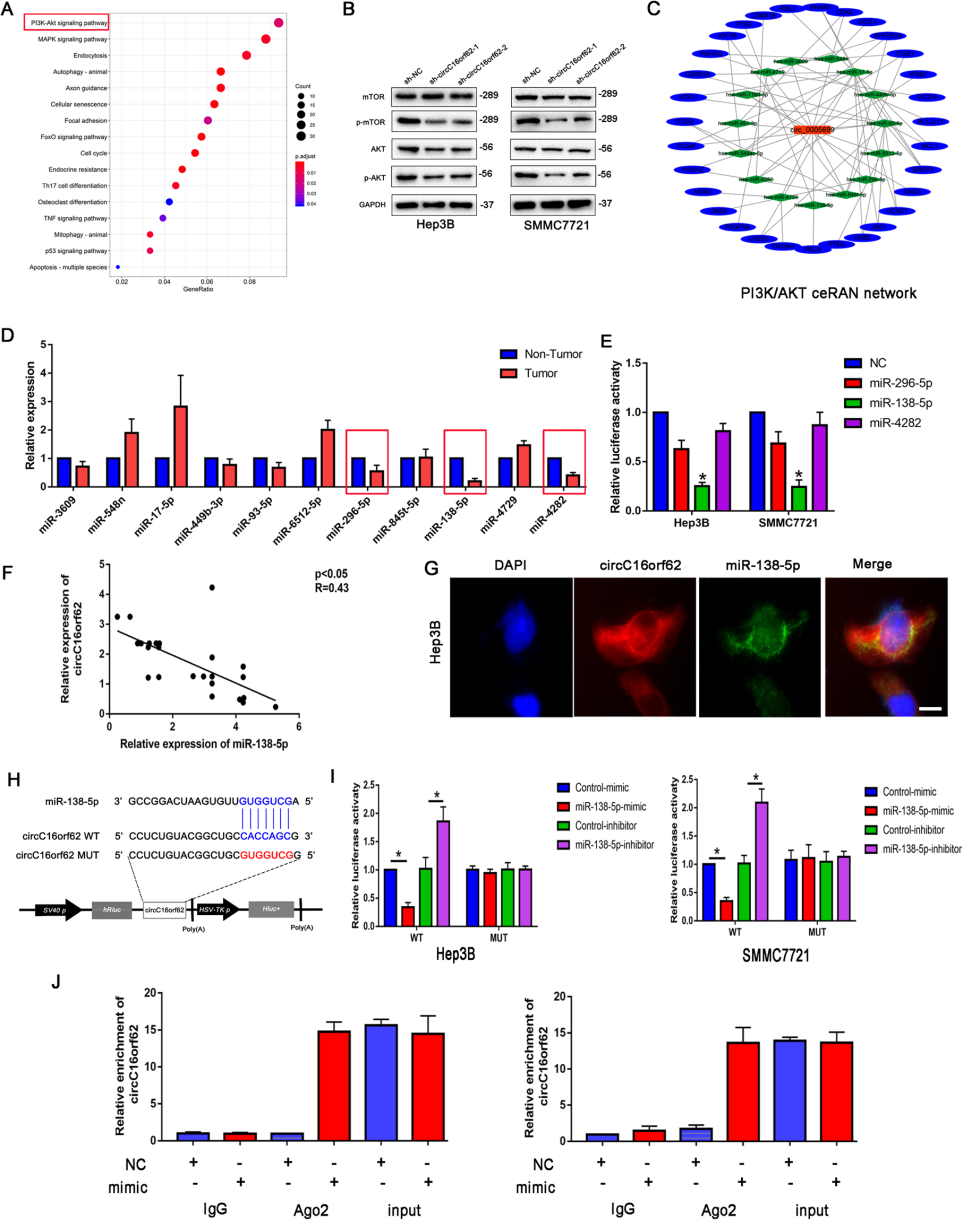
Bioinformatic analysis and validation of potentially targeted miRNAs of circC16orf62 in HCC cells. **A** KEGG pathway analysis the downstream signal pathway eluted the PI3K/AKT was the significantly downstream pathway of circC16orf62. **B** Western-blots analysis the expression of p-AKT and p-mTOR. **C** ceRNA network analysis the target miRNAs of circC16orf62. **D** RT-qPCR and western blot analysis the target miRNAs in the HCC tumor and adjacent normal tissue. **E** Luciferase analysis the interaction of circC16orf62 and miRNA-138. **F** RT-qPCR analysis the expression correlation of circC16orf62 and miRNA-138 in the HCC tumor and adjacent normal tissue. **G** FISH assay analysis the circC16orf62 and miRNA-138 co-location in the Hep3B cell, the scale bar represents 10 µm. **H** TargetScan showed that circC16orf62 could bind to the miR-138-5p. **I** The direct binding between circC16orf62 and miRNA-138 was analyzed by dual-luciferase reporter assay. **J** A RIP assay was performed using an AGO2 antibody, and IgG served as a negative control. Data are shown as the mean ± standard deviation of three independent experiments.*P < 0.05was regarded as statistically significant.

To figure out the role of circC16orf62, we made the prediction on the potential targeting miRNAs in CSCD (http://gb.whu.edu.cn/CSCD/) database and target genes(starbase) by bioinformatics. Furthermore, KEGG pathway analysis the downstream signal pathway eluted the PI3K/AKT was the significantly downstream pathway of circC16orf62 (Fig. 4A). Then, the western-blots result demonstrated that circ C16orf62 knockdown could make inhibition on the p-AKT and p-mTOR expression (Fig. 4B). Based on the PI3K/AKT pathway, we constructed the ceRNA regulation network to select the targeted miRNAs (Fig. 4C). Among the 15 miRNAs, miRNA-296-5p, miRNA-138-5p, and miRNA-4282 were significantly downregulated in the HCC tissues (Fig. 4D). Further, the luciferase activity assay indicated that the miRNA-138 could inhibit the luciferase activity of circC16orf62 3′UTR (Fig. 4E). The q-RT-PCR assay detected the expression of circC16orf62 and miR-138-5p, Person analysis the expression level illustrated that the circRNA and miRNA mentioned were negatively correlated in the HCC tissues (Fig. 4F). To verified the location of the two circRNAs, the FISH assay pointed out that two types of circRNA were mainly co-located in the cytoplasm (Fig. 4G). Bioinformation analysis the binding site of the two, the corresponding mutation site was shown in Fig. 4H. The luciferase activity assay indicated that miR-138-5p could combine with circC16orf62 (Fig. 4I). Then, the RIP assay indicated that Ago2 could combine with the two, the results indicated that they were acted on the Ago2 (Fig. 4J). Together, the results above show that circC16orf62 perhaps sponge miR-138-5p in HCC cells.

### MiRNA-138-5p targets the PTK2 expression involved in AKT/mTOR signal pathway

**Fig. 5:**
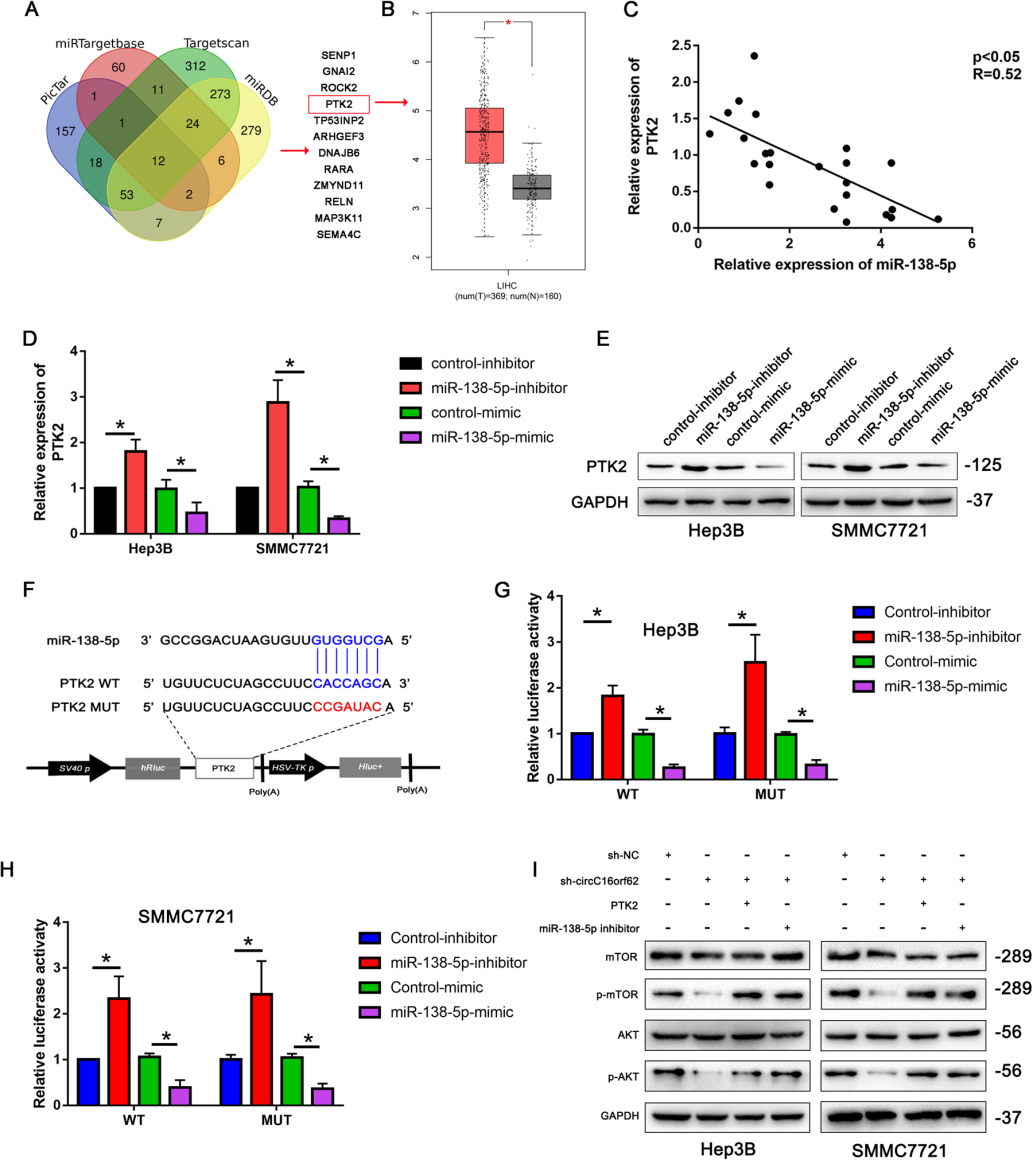
MiRNA-138-5p targets the PTK2 expression involved in AKT/mTOR signal pathway. **A** Bioinformatic analysis of the potential target gene by prediction software(PicTar, miRTargetbase, Targetscan, miRDB). **B** TACG data base analysis of the expression level of PTK2 in HCC. **C** The person analysis the miRNA-138-5p and PTK2 expression correlation in HCC tissues. **D**, **E** The q-RT-PCR and western-blot assay analysis the PTK2 expression in miRNA-138-5p inhibition and overexpressed HCC cells. **F** The bioinformation analysis the binding site of miRNA-138-5p and PTK2. **G**, **H** The luciferase activity assay was used to verify the combination of miRNA-138-5p and PTK2. **I** The western blot analysis miRNA-138-5p targets the PTK2 expression involved in PI3K/AKT signal pathway. Data are shown as the mean ± standard deviation of three independent experiments. *P < 0.05was regarded as statistically significant.

The target genes with the potentiality of miRNA-138-5p were picked out by the prediction software(PicTar, miRTargetbase, Targetscan, miRDB). Based on the bioinformatic analysis, PTK2 was found as the putative target gene (Fig. 5A and Supplementary Material 1). Based on the TCGA database, the results indicated that PTK2 was upregulated in the HCC (Fig. 5B). Figure 5C shows the person analysis demonstrated that miRNA-138-5p and PTK2 were negatively correlated in HCC tissues. Furthermore, the q-RT-PCR and western-blot assay indicated that miRNA could inhibit that PTK2 expression in HCC cells (Fig. 5D, E). Then, the bioinformation analysis found the binding site of miRNA-138-5p and PTK2, the luciferase wide type and mutation type plasmid was constructed according to the binding site (Fig. 5F). The luciferase activity assay was used to verify the combination of miRNA-138-5p and PTK2, the result indicated that miRNA-138-5p could bind with the PTK2 3′ UTR (Fig. 5G, H). Then, the western blots indicated that circC16orf62 knockdown inhibited AKT/mTOR signaling pathway, and the effect could partly be inhibited by miR-138 inhibitor or PTK2 (Fig. 5I). Data above indicated that PTK2 was the potential function target of miRNA-138-5p in the progress of HCC.

### circC16orf62 knockdown repressed HCC progression via controlling MiR-138-5p/PTK2

**Fig. 6:**
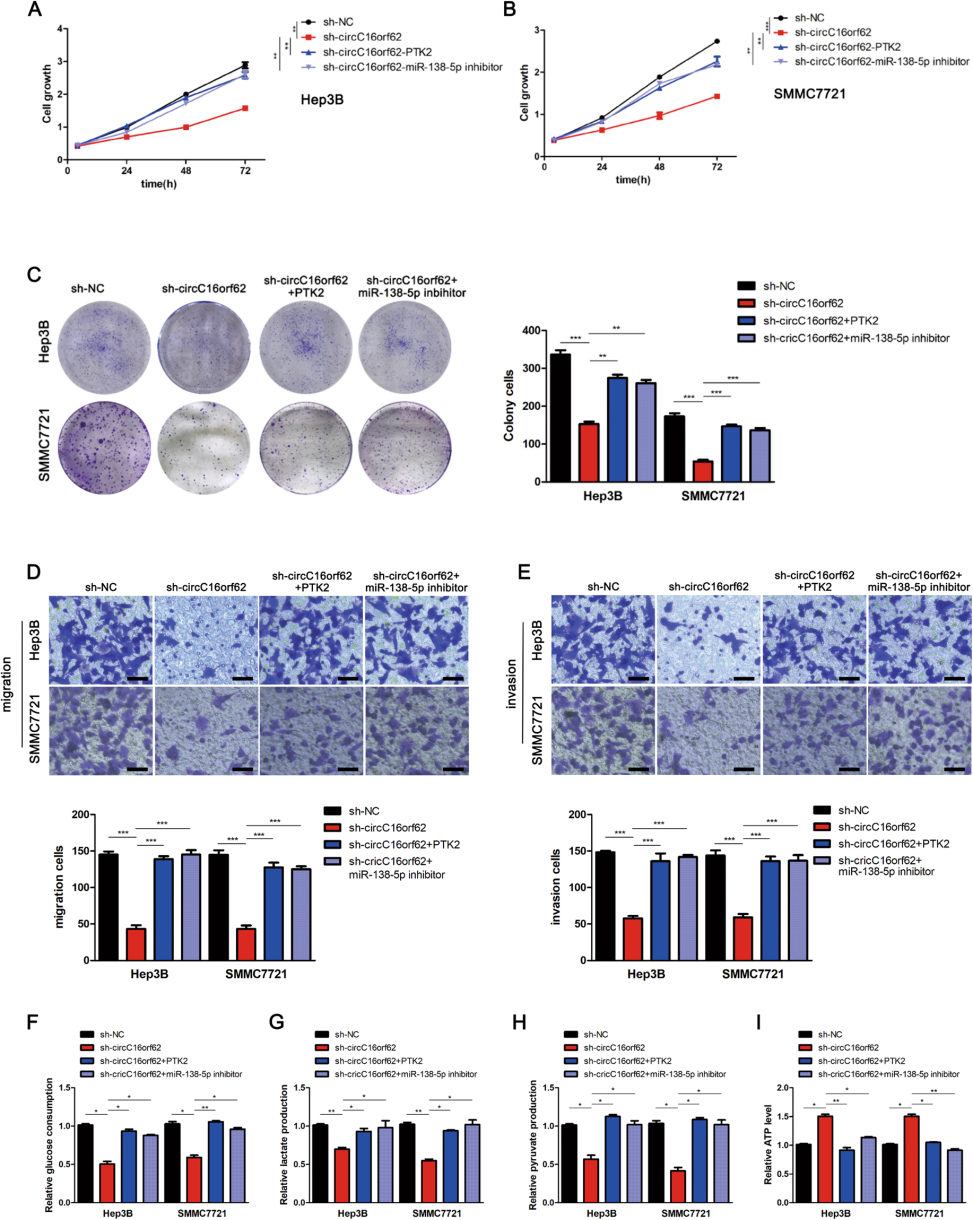
circC16orf62 knockdown repressed HCC progression via regulating MiR-138-5p/PTK2. **A**–**C** Cell proliferation of four indicated groups was individually tested by CCK8 and plate formation assays. **D**, **E** Transwell assay was carried out for detecting cell migration and invasion in different groups, the scale bar represents 50 µm. **F**–**I** Glucose consumption (**F**), lactate production (**G**), pyruvate production (**H**), ATP production (**I**) experiments were conducted to estimate cell motility in three groups. Data are shown as the mean ± standard deviation of three independent experiments. *P < 0.05 was regarded as statistically significant.

HCC cells were transfected with sh-circC16orf62, and co-transfected with miR-138-inhibitor or PTK2 overexpressed plasmid. The CCK8 and plate colony assay indicated that circC16orf62 could promote the growth ability of HCC, and miR-138 inhibitor and PTK2 overexpress plasmid could partly reverse the effect of circC16orf62 (Fig. 6A–C). And the transwell assay indicated that the migration and invasion of HCC cells were upregulated in the cell that transfected with miR-138-inhibitor or PTK2 overexpressed plasmid (Fig. 6D, E). Then, MiR-138-5p/PTK2 could increase glucose consumption (Fig. 6F), lactate production (Fig. 6G), pyruvate production (Fig. 6H), and decrease ATP level in the HCC cells transfected with circC16orf62 shRNA (Fig. 6I). These data eluted that circC16orf62 knockdown repressed HCC progression via regulating MiR-138-5p/PTK2.

### Knockdown of circC16orf62 shows growth inhibition of HCC cell in vivo

**Fig. 7:**
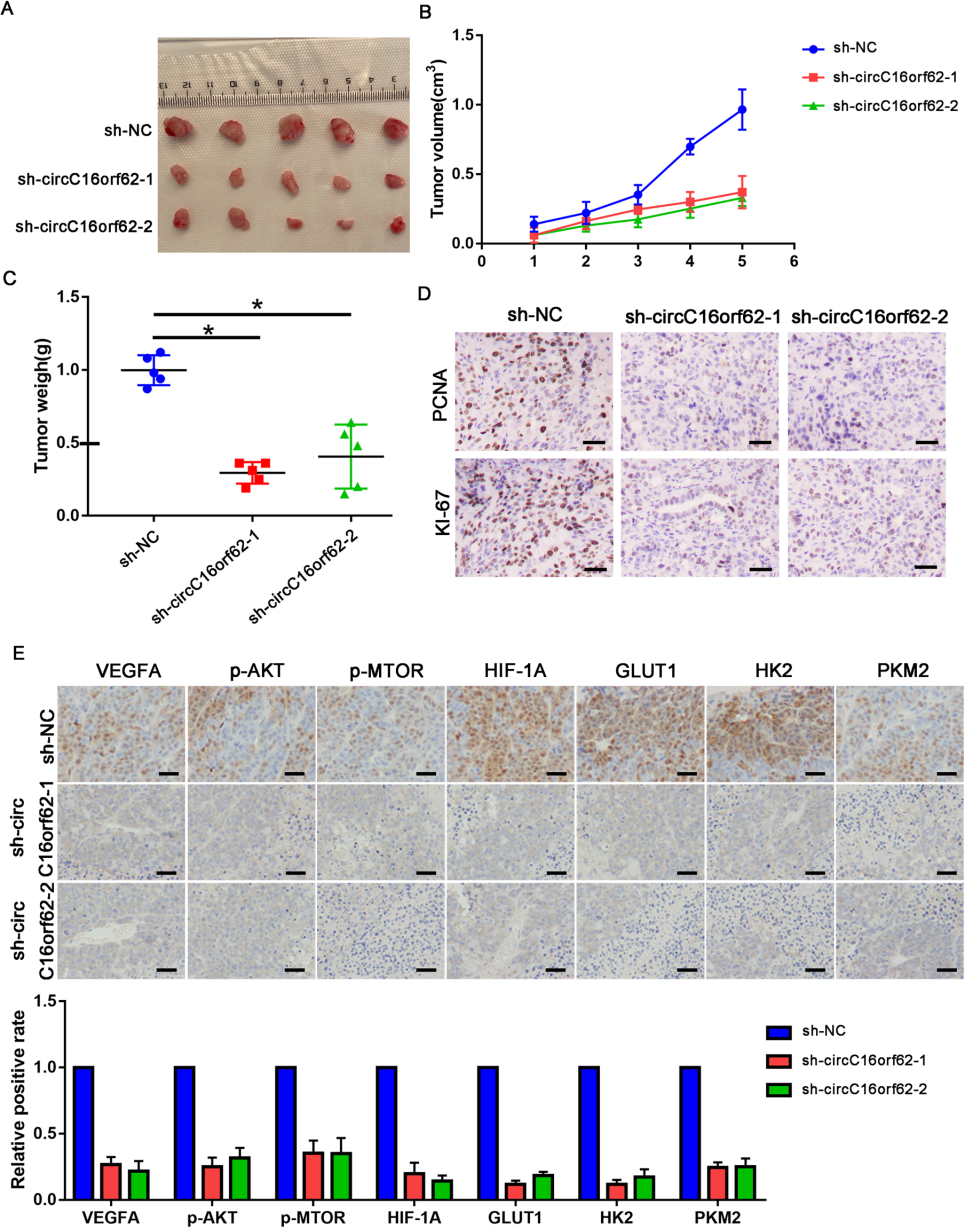
Knockdown of circC16orf62 inhibited in vivo growth of HCC cell. **A** The BALB/c nude mice were sacrificed for the xenografts, and the size was measured by the beside ruler. **B** The tumor growth curve of xenografts was plotted in shNC and shcircC16orf62 group (*n* = 5 each group) by measuring the tumor size (width^2^ × length × 0.5) with vernier caliper. **C** The anatomized subcutaneous tumor xenografts were weighed and analyzed with student’s *t* test. **D** IHC analysis the proliferation markers (PCNA and KI-67) in different group xenografts, the scale bar represents 100 µm. **E** IHC analysis the AKT/mTOR signaling pathway markers in different group xenografts, the scale bar represents 100 µm. Data are shown as the mean ± standard deviation of three independent experiments. *P < 0.05 was regarded as statistically significant.

To figure out the role of circC16orf62 in HCC growth in vivo, we injected Hep3B cells transfected with NC or circC16orf62 shRNAs(sh-circC16orf62-1, sh-circC16orf62-2) to the mice. Knockdown of sh-circC16orf62 reduced the weight of the tumor after 5-week subcutaneous injection (Fig. 7A–C). KI-67 and PCNA staining assured that lower growth ability presents in the mice which were with SMMC7721 cells transfected with sh-circC16orf62 shRNA (Fig. 7D). IHC detects positive expression of PTK2, p-AKT, p-MTOR, PKM2, GLUT1, HK2. And expression levels of the target gene PTK2, the AKT signal indicator p-AKT, and p-MTOR, the glycolysis target PKM2, GLUT1 and HK2 were downregulated through the transfection of circC16orf62 shRNA (Fig. 7E). All in all, circC16orf62 serves as an activator of osteosarcoma progression in vivo.

## Discussion

HCC cancer is the commonest malignant tumor that patients has increased year by year^18^. Endoscopic minimally invasive surgery and surgical eradication can be used to deal with early HCC^19,20^. For no significant symptoms of most cases, almost 60% of HCC has progressed when diagnose with lymph node and distant metastases (into the middle and late stages)^21^. Early detection and treatment lead to a better prognosis. For that, finding an effective novel biomarker and exploring HCC pathogenesis-related signaling pathways is vital^22,23^.

Based on the GEO database, we analysis the different circRNAs in seven pairs HCC tissue and corresponding adjacent normal tissues. We figure out the differently expressed circRNAs in HCC tissues and corresponding adjacent normal tissues. The circRNAs could be biomarkers with potentiality and therapeutic targets for the diagnosis HCC. Based on our data, selected circRNAs exhibit vital distinguishment in expression and they were measured in additional samples. The expression level of it was obviously related to clinical stage and malignancy. survival analysis pointed out that circC16orf62 in HCC may be a promising prognostic biomarker. Various functional experiments suggested that knockdown of the circRNA mentioned vitally inhibits the proliferation and invasion of the cancer cells, as the mentioned one functions likely an oncogene. Especially, the circC16orf62 expression was obviously enhanced in the HCC bigger tumor size and distal metastasis group. When taking the effect on the invasion of HCC cells in the human body into consideration, circC16orf62 functions vital in the malignant progression.

The miRNA sponge mechanism is a foundation for detecting the bio-functions of circRNAs^24^. Accumulated literature discovered that circRNA could function as a miRNA sponge. The RIP assay confirmed that circC16orf62 adsorbs miRNA. So we used target prediction software to predict circRNA-miRNA-mRNA interactions through and built associated networks. Dual-luciferase reporter assays found that circC16orf62 could directly interact with miR-138-5p (predicted target). The rescue experiments indicated that decreased HCC cell proliferation and invasion resulted from circC16orf62 knockdown offset for miR-138-5p inhibition. A report indicated that this miRNA functions like a tumor suppressor in many cancers^25,26^, and the interaction of the two attenuates the tumor suppressor efficiency of miR-138-5p. circC16orf62 is like an oncogene, sponging miR-138-5p in HCC.

PI3K/AKT signaling is widely activated in human cancers, and investigation on it has been well performed^27^. In addition, some cancers contain overexpression of PTK2 which reported function as oncogene^28^. PTK2 mRNA and protein expression in HCC tissues were higher in our data than those in adjacent normal tissues which vital positive relationship with the expression of circC16orf62. The function assay indicated that PTK2 acted as an activator to assist the promoting effect exerted by circC16orf62 in HCC cell proliferation and invasion. Meanwhile, PTK2 was known as a protein kinase to activte AKT/mTOR signaling pathway, Can play a regulatory role in the occurrence and development of tumors. Function recovery experiment found that the miR-138-5p inhibitor partly restrained the effect of circC16orf62 inhibition on cell proliferation. Abnormal activation of AKT-mTOR signaling pathway functions vital in the malignant progression of HCC. The results indicated that downregulation of circC16orf62 noticeably inhibit the expression level of PTK2 which further mediating AKT/mTOR signaling activating in HCC. So the circC16orf62 /miR-138-5p/PTK2/AKT signaling pathway functions vital in HCC. In conclusion, circC16orf62, a biomarker with much potential, is upregulated human HCC issues. The effect of the circC16orf62/miR-138-5p/PTK2/AKT regulation network on the progress and deterioration of HCC. We believe that circC16orf62 is a therapeutic target with potential for HCC.

## Supplementary information

Supplemetary Material 1

Supplementary figure and table legends

## Data Availability

All data generated and analysed during this study are included in this published article are available on request.
