# Supplementary figures and images for: CircC16orf62 promotes hepatocellular carcinoma progression through the miR-138-5p/PTK2/AKT axis
Source: Cell Death Dis. 2021 Jun 9;12(6):597. doi: 10.1038/s41419-021-03866-7 (PMC8190090; doi:10.1038/s41419-021-03866-7)

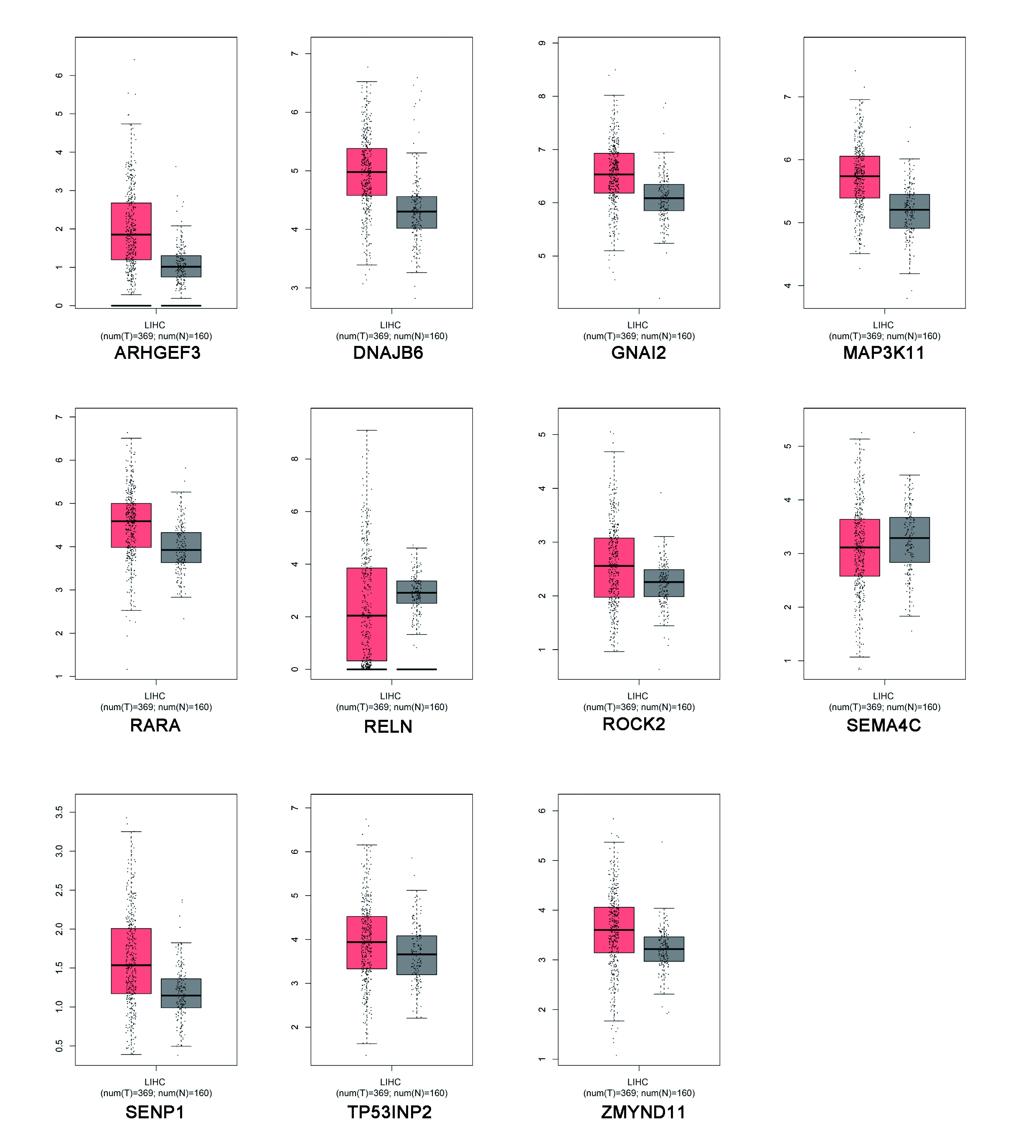

Supplement: Supplementary file 1 — Supplemetary Material 1 [file 41419_2021_3866_MOESM1_ESM.tif]
